# An extended target tracking algorithm based on trajectory feedback constrained spatial information

**DOI:** 10.1371/journal.pone.0357813

**Published:** 2026-09-18

**Authors:** Yuting Zhang, Hantao Li, Jiye Li

**Affiliations:** 1 The School of Computer Engineering, Chengdu Technological University, Chengdu, SiChuan, P.R. China; 2 The School of Artificial Intelligence, Chongqing University of Education, Chongqing, P.R. China; Northwest Normal University, CHINA

## Abstract

Accurately estimating the state of extended targets is a major challenge because measurement numbers and distributions change significantly, especially when tracking targets are close or overlapping. To solve the track fragmentation problem caused by spatial measurement ambiguity, a novel Trajectory Situation Feedback-based Gaussian Mixture Model Expectation Maximization (TSF-GMM-EM) method is proposed within the framework of the Gaussian Mixture Probability Hypothesis Density (GM-PHD) filter. The proposed method feeds historical trajectory information back to guide current measurement partitioning and motion estimation while providing prior-guided initialization for the EM process, thereby improving partitioning reliability during target intersections and accelerating clustering convergence. Experimental results show that the TSF-GMM-EM method limits the peak OSPA error to approximately 12 m during target intersections. It also maintains a measurement partitioning accuracy above 0.946 in overlapping target scenarios.

## 1. Introduction

Multiple target tracking (MTT) has been used to track multiple targets using various sensors [1]. Point target tracking (PTT) and extended target tracking (ETT) are fundamental components in numerous real-world applications [2–4]. With the advancement of high-resolution sensors [5–9], an extended target typically generates multiple measurements per scan. These multiple measurements allow for the estimation of additional target states, such as shape and orientation [10,11]. However, the presence of false alarms [12] and the increasing number of measurements introduce significant challenges to multi-extended target tracking (MTT). Specifically, the complexity of data association is exacerbated when measurement numbers and locations are time-varying, or when targets are in close proximity or overlapping. In such scenarios, incorrect measurement partitioning leads to degraded tracking accuracy or even track loss. Moreover, estimating target motion states from noisy measurements remains a fundamental challenge, as clutter interference inherently degrades the precision of state estimation [13,14]. Therefore, effective measurement partitioning remains a critical issue for enhancing MTT performance.

To address the inherent complexities of multi-target tracking problems, the Random Finite Set (RFS) framework has been extensively adopted, different from traditional data association-based methods, such as Joint Probabilistic Data Association (JPDA) filter and multiple hypotheses tracking (MHT). However, these methods incur a significant computational overhead as the number of targets and measurements increases. By modeling both target states and measurements as random sets, the RFS-based method effectively circumvents the combinatorial explosion associated with explicit data association [15–17]. A cornerstone of this framework is the Probability Hypothesis Density (PHD) filter proposed by Mahler [18,19], which propagates the first-order moment of the multi-target posterior density. Although the original Poisson-based recursions provided a solid theoretical foundation, their implementation remained computationally prohibitive for real-time applications until the development of closed-form solutions like the Gaussian Mixture (GM-PHD) filter. To maintain computational efficiency, the GM-PHD filter incorporates pruning and merging operations that effectively constrain the recursive expansion of Gaussian components. By eliminating explicit data association, the GM-PHD framework delivers reliable tracking performance with minimal computational cost.

Over the past decades, the advancement of Extended Target Tracking (ETT) has evolved through several distinct methodology paradigms, primarily focusing on how to jointly estimate target kinematics and spatial extensions [20,21]. Early pioneered works heavily relied on spatial shape parameterization, most notably the Random Matrix (RM) approach introduced by Koch [11], which models the target extension as a positive-definite matrix following an inverted Wishart distribution. This framework was later extended to multi-target scenarios by Granström et al. [22] and adapted for maneuvering non-ellipsoidal shapes by Liang et al. [3] using sub-random matrices. Concurrently, non-parametric approaches such as Monte Carlo methods [10], alongside spatiotemporal Gaussian Processes (GP) [23,24], were introduced to approximate highly irregular geometric contours.

To bridge the gap between contour tracking and intent inference, recent many researchers have shifted toward Joint Tracking and Classification (JTC). Notably, Cheng et al. [25] proposed an extended target JTC filter based on GPs and the Labeled Multi-Bernoulli (LMB) framework, while class-enhanced GP-PHD variants [26] optimized rectangular constraints for complex vehicle profiles. More recently, to explicitly capture multi-frame temporal tracks, the Trajectory Probability Hypothesis Density (TPHD) filter was integrated with Random Matrix Models [27], enabling the simultaneous recursive estimation of target states, classes, and explicit trajectory sets. While these filtering frameworks offer solid mathematical solutions for shape tracking, their state recursion accuracy remains fundamentally bounded by the data association layer, specifically the quality of measurement partitioning in dense clutter environments. Moreover, Al-Haddad et al. [28] have significantly elevated the robustness of trajectory control against non-linear disturbances, and it is crucial to connect these filtering breakthroughs with state-of-the-art autonomous applications.

To feed the multi-target RFS update recursions—such as the Extended Target GM-PHD filter [22] and the interactive multiple-model (IMM-PHD) filter for coexisting point and extended targets proposed by Li et al. [29]—measurement partitioning acts as a critical front-end clustering mechanism that groups unlabelled point clouds into individual target-originated cells. Existing partitioning strategies can be broadly classified into two categories: spatially-driven heuristics and statistical mixture optimization models.

Within the spatially-driven category, Distance Partitioning (DP) [22] and Density-Based Spatial Clustering (DBSCAN) [30] are widely utilized due to their intuitive implementation based on spatial proximity. However, DP suffers from severe computational overhead because it necessitates iterating through an extensive range of predefined distance thresholds to handle varying target ranges [31], frequently generating redundant partitions. Similarly, DBSCAN is notoriously sensitive to its hyper-parameters, exhibiting poor robustness when dealing with heterogeneous target sizes. To alleviate threshold sensitivity, scalable association-based non-homogeneous Poisson process (AbNHPP) frameworks [32] have emerged in 2026 to dynamically model time-varying measurement rates using Generalized Inverse Gaussian distributions.

To circumvent the rigid thresholding of heuristic partitioning, optimization-based soft clustering approaches utilizing Gaussian Mixture Models and the Expectation-Maximization (GMM-EM) algorithm have been introduced [33,34]. These methods partition measurements based on continuous posterior association probabilities rather than hard spatial boundaries. Nevertheless, standard GMM-EM formulations operate purely in the spatial domain of a single time-frame, leaving them highly vulnerable to the structural initializations of cluster centers. Consequently, in complex scenarios where multiple extended targets closely approach, merge, or overlap, the standard EM iteration inherently traps into uninformative local maxima [20]. This directly manifests as severe cardinality estimation errors, track fragmentation, or catastrophic track loss.

Existing partitioning strategies, such as distance partitioning (DP) [22] and DBSCAN [30], rely on the spatial closeness of measurements. However, DP often requires predefined distance thresholds, which can lead to redundant partitions and high computational costs [31]. Similarly, DBSCAN’s performance is sensitive to its radius and minimum-point parameters, showing poor robustness in environments with diverse target types. Alternatively, the Expectation-Maximization (EM) algorithm and Gaussian Mixture Models (GMM) [33,34] partition measurements based on posterior probabilities. Nevertheless, standard GMM-EM often converges to local maxima, resulting in uninformative partitions—especially when targets are close or converging.

A key limitation of the aforementioned methods is their reliance on spatial information from a single time step. In reality, target features exhibit strong spatio-temporal correlations across frames [23,24,35]. Exploiting the target’s trajectory—which inherently contains prior information about target numbers and measurement structures—could break the current bottleneck in partitioning and state estimation.

### 1.1 Contributions

Based on the GM-PHD filter, this paper proposes a Trajectory Situation Feedback-based Gaussian Mixture Model Expectation Maximization (TSF-GMM-EM) method. The main contributions and advantages are summarized as follows:

- **A closed-loop trajectory feedback framework:** We establish a bidirectional interaction between trajectory evolution and measurement partitioning. Different from the open-loop methods, the proposed TSF-GMM-EM leverages temporal trajectory priors to guide spatial partitioning, effectively resolving data association ambiguity in complex scenarios.
- **A prior-guided EM initialization strategy:** By leveraging predicted target states to drive the measurement partitioning process, this strategy ensures spatio-temporal consistency between target trajectories and measurement clusters. Moreover, this method effectively avoids local optima in the GMM-EM framework and prevents cardinality errors during target intersections, thereby enhancing both tracking precision.

### 1.2 Organization of the paper

The remainder of this paper is structured as follows. Section 2 establishes the extended target tracking mathematical model within the Random Finite Set framework and details the proposed TSF-GMM-EM method, including trajectory-guided measurement partitioning and intersection situation judgment. Section 3 presents the comprehensive experimental framework. Finally, Section 4 concludes the paper and outlines potential directions for future research.

## 2. Method

In this section, the extended target tracking problem is formulated within the Random Finite Set model. The Gaussian Mixture Probability Hypothesis Density filter is adopted as the baseline, where the target states and measurements are modeled as RFS to bypass explicit data association. For extended targets, the update step of the GM-PHD filter relies heavily on the quality of measurement partitioning. However, traditional partitioning methods often fail to provide accurate subsets when targets are in close scene. To address this, a feedback mechanism is introduced to bridge the gap between trajectory estimation and measurement clustering.

### 2.1 Model

A number of measurements from an extended target spread over a region at every instant, and their distribution changes over time [36]. In essence, multi-extended targets tracking is to estimate the number of targets and every target’s state $X_{k}={\left\{x_{k}^{\left(i\right)}\right\}}_{i=1}^{N_{x,k}}$ from the measurements $Z_{k}={\left\{z_{k}^{\left(i\right)}\right\}}_{i=1}^{N_{z,k}}$ up to instant *k*, where $N_{x,k}$ denotes the unknown number produced by targets and $N_{z,k}$ denotes the available number of measurements. The dynamic evolution of each target state $x_{k}^{\left(i\right)}$ in the RFS $X_{k}$ can be modeled by a linear Gaussian dynamic model, and its motion state equation could be denoted as


$$\begin{array}{c} x_{k+1}^{\left(i\right)}=T_{k}x_{k}^{\left(i\right)}+G_{k}w_{k}^{\left(i\right)} \end{array}$$ (1)

where $T_{k}$ and $G_{k}$ are the known state transition matrix, $w_{k}^{\left(i\right)}$is Gaussian white noise with covariance matrix $Q_{k}^{\left(i\right)}$. It is worth noting that each target state is independent between others.

The number of measurements generated by an extended target is assumed by a Poisson-distributed random variable with rate $\gamma \left(x_{k}^{\left(i\right)}\right)$ at every instant, where γ(·) is a known non-negative function. For an extended target, the detection probability could be defined as


$$\begin{array}{c} P_{D,eff}=\left(1-e^{-\gamma \left(x_{k}^{\left(i\right)}\right)}\right)P_{D}\left(x_{k}^{\left(i\right)}\right) \end{array}$$ (2)

where $P_{D}\left(x_{k}^{\left(i\right)}\right)$ denotes the probability of i−th target, where $P_{D}\left(\cdot \right)$ is a known non-negative function.

The measurements originating from the i−th target are usually modeled as


$$\begin{array}{c} z_{k}^{\left(i\right)}=H_{k}x_{k}^{\left(i\right)}+e_{k}^{\left(i\right)} \end{array}$$ (3)

where $H_{k}$ is a known measurement matrix, and $e_{k}^{\left(i\right)}$ denotes white Gaussian noise with covariance matrix $R_{k}^{\left(i\right)}$. The noise vectors $w_{k}^{\left(i\right)}$ and $e_{k}^{\left(i\right)}$ are statistically independent.

In addition to the measurements generated by the extended targets, there are also false alarm in the measurement space. The number of false alarm measurements at instant *k* is also a Poisson distributed random variable with rate $\beta_{FA,k}$, and the mean number of false alarm measurements is $\beta_{FA,k}V_{S}$, where $V_{S}$ is the surveillance volume. Moreover, the false alarm measurements is assumed as uniform distribution in the spatial domain.

In the ET-GM-PHD filter, the measurement update formula can be represented as


$$\begin{array}{c} D_{k|k}\left(x|Z\right)=L_{Z_{k}}\left(x\right)D_{k|k-1}\left(x|Z\right) \end{array}$$ (4)

where the pseudo-likelihood function $L_{Z_{k}}$ is defined as


$$\begin{array}{cc} L_{{𝐙}_{k}}(𝐱)= & 1-\left(1-e^{-\gamma (𝐱)}\right)P_{D}(𝐱)\\ & +e^{-\gamma (𝐱)}P_{D}(𝐱)\times \sum_{p∠{𝐙}_{k}}\omega_{p}\sum_{W\in p}\frac{\gamma (𝐱{)}^{\left|W\right|}}{d_{W}}\\ & \cdot \prod_{z\in W}\frac{\phi_{{𝐳}_{k}}(𝐱)}{\lambda_{k}c_{k}({𝐳}_{k})} \end{array}$$ (5)

where $\phi_{z_{k}}\left(x\right)=p\left(z_{k}|x\right)$ is the likelihood function of the measurement generated from a single target, $\lambda_{k}$ denotes the mean number of false alarm measurements, $c_{k}\left(z_{k}\right)=1/V_{S}$ denotes the spatial distribution of the false alarm over the surveillance volume, $p∠Z_{k}$ denotes the sub-set *p* of measurement $Z_{k}$ by means of measurement partition, and each nonempty cells *W* contains a potential measurement result of the extended target.

### 2.2 The proposed method

Inspired by the strong short-term spatio-temporal correlations exhibited by extended target measurements and motion trajectories, the proposed method leverages temporal information from previous frames to guide the current spatial partitioning. Specifically, it is structured into two primary modules: trajectory-guided measurement partitioning and intersection situation judgment.

#### 2.2.1 Trajectory-guided measurement partitioning.

The core idea of the expectation maximization is to find certain parameter for the complete data by maximizing the expectation likelihood [28]. The Gaussian mixture model EM (GMM-EM) algorithm estimates the probability that a measurement belongs to each cluster by the GMM parameters [37,38].


$$\begin{array}{c} p\left(z^{\left(i\right)}|\theta \right)=\sum_{c=1}^{C}\pi_{c}N\left(z^{\left(i\right)}|\mu_{c},P_{c}\right) \end{array}$$ (6)

where $z^{\left(i\right)}$ is the measurements, $\theta ={\left\{\pi_{c},\mu_{c},P_{c}\right\}}_{c=1}^{C}$ denotes the parameters of GMM, *C* is the number of Gaussian sub-models, $\mu_{c}\in {\mathbb{R}}^{2x1}$ and $P_{c}\in {\mathbb{R}}^{2x2}$ are the mean vector and the covariance matrix of the c−th Gaussian component, respectively. Moreover, $\pi_{c}$ is the component weight of the c−th Gaussian component, and it abides by $0<\pi_{c}\leq 1$ and $\sum_{c=1}^{C}\pi_{c}=1$.

In order to simplify the calculation, the log calculation L(θ) is performed on the maximum likelihood function to search the optimal parameters $\theta^{*}$.


$$\begin{array}{c} L\left(\theta \right)=\sum_{i=1}^{N_{z}}\log\left(p\left(z^{\left(i\right)}|\theta \right)\right) \end{array}$$ (7)

$$\begin{array}{c} \theta^{*}=\underset{\theta }{\text{arg}\max}L\left(\theta \right) \end{array}$$ (8)

where $N_{z}$ is the amount of all measurements, and the symbol argmax denotes maximizing the likelihood function to get the optimal parameters $\theta^{*}$.

However, conventional GMM-EM typically operates in a purely spatial domain, often neglecting the temporal continuity of target motion. To bridge this gap, the proposed method extends the conventional likelihood maximization framework into the spatio-temporal domain to achieve a more robust partition. Specifically, let ${𝐅}_{k}$ represent the temporal series information at instant *k*, encompassing the target count, motion states, and measurement distributions. Assuming that the target’s characteristic information remains quasi-stationary over short intervals, TSF-GMM-EM identifies the optimal parameter set $\theta^{*}=\{\pi_{k}^{*},\mu_{k}^{*},{𝐏}_{k}^{*}\}$ by maximizing the likelihood function conditioned on the temporal series ${𝐅}_{k}$. Here, the optimal parameters $\theta^{*}$ denote the estimated Gaussian components of multiple extended targets. Consequently, the historical information ${𝐅}_{k-1}$ accumulates the optimal estimation parameters up to instant k−1, which can be formally expressed as:


$$\begin{array}{c} F_{k-1}=\left\{\theta_{1}^{*},\theta_{2}^{*},...,\theta_{k-1}^{*}\right\} \end{array}$$ (9)

As the covariance of an extended target, $P\in {\mathbb{R}}^{2x2}$ represent the shape information of Gaussian surface, and it is denoted as


$$\begin{array}{c} P=\left[\begin{array}{cc} \sigma_{xx}^{2} & \sigma_{xy}^{2}\\ \sigma_{yx}^{2} & \sigma_{yy}^{2} \end{array}\right] \end{array}$$ (10)

where $\sigma_{xx}^{2}$ and $\sigma_{yy}^{2}$ are the variances at horizontal direction and vertical direction variance, respectively, and $\sigma_{xy}^{2}=\sigma_{yx}^{2}$ represents the variance in horizontal biased vertical direction.

Normally, the state of an extended target including the measurements and the motion is consistent and correlative until multiple targets approach and encounter during tracking process. Meanwhile, all measurements usually are disturbed by noise. Therefore, TSF-GMM-EM assumes the estimated parameters ${\hat{P}}_{c}^{*}$ obeys a one-dimensional Gaussian distribution as


$$\begin{array}{c} {\hat{P}}_{c}^{*}\left(k\right)\sim 𝒩\left({ℳ}_{c},\sigma_{c}^{2}\right),c=1,2,...,C \end{array}$$ (11)

where ${ℳ}_{c}$ and $\sigma_{c}^{2}$ denotes the mean and the variance of the c−th target’s true covariance, respectively; ${{\hat{P}}^{*}}_{c}(k)$ denotes the estimated optimal feature of c−th target at instant *k*.

It is widely known that the states including approaching and meet of multiple extended targets can be predicted in advance by means of their trajectory. Therefore, it is possible to accurately estimate the measurements partition for extended targets by means of predicting the state even at the situation that their measurements are mixed up when they approach or encounter. When the situation that multiple targets approach or encounter is predicted, TSF-GMM-EM would use predicted state to constrain the measurements partition as:


$$\begin{array}{c} P_{c}^{*}=\frac{1}{k-1}\sum_{i=1}^{k-1}{\hat{P}}_{c}^{*}\left(i\right),S\in \text{Encounter} \end{array}$$ (12)

where S∈Encounter denotes that multiple targets encounter. Statistically, when spatial measurements collapse into extreme ambiguity during close encounters, the current-frame observational likelihood becomes highly non-informative. Under the hypothesis of short-term temporal quasi-stationarity of target trajectories, the formulation in Eq. (12) serves as a localized empirical expectation estimator. By leveraging the arithmetic sample mean over a short historical window preceding the intersection, this mechanism functions as a Minimum Mean Square Error (MMSE) predictor that minimizes individual frame noise variance, thereby providing a stable, time-continuous prior constraint to regularize the soft-clustering optimization.

For the Expectation step (E-step), we have the posterior probability $\alpha_{c}^{\zeta +1}\left(z^{\left(i\right)}\right)$ oriented from the parameters $\theta^{\zeta }={\left\{{\hat{\pi }}_{c}^{\zeta },{\hat{\mu }}_{c}^{\zeta },{\hat{P}}_{c}^{\zeta }\right\}}_{c=1}^{C}$ as follows:


$$\begin{array}{c} \alpha_{c}^{\zeta +1}\left(z^{\left(i\right)}\right)=\frac{{\hat{\pi }}_{c}^{\zeta }\cdot N\left(z^{\left(i\right)}|{\hat{\mu }}_{c}^{\zeta },{\hat{P}}_{c}^{\zeta }\right)}{\sum_{c=1}^{C}{\hat{\pi }}_{c}^{\zeta }N\left(z^{\left(i\right)}|{\hat{\mu }}_{c}^{\zeta },{\hat{P}}_{c}^{\zeta }\right)} \end{array}$$ (13)

where ${\hat{\pi }}_{c}^{\zeta }$, ${\hat{\mu }}_{c}^{\zeta }$ and ${\hat{P}}_{c}^{\zeta }$ denotes the estimated weight, mean vector and covariance matrix of c−th Gaussian sub-model at step ζ, respectively. The maximization step (M-step) of TSF-GMM-EM method can be denoted as


$$\begin{array}{c} {\hat{\pi }}_{c}^{\zeta +1}=\frac{\sum_{i=1}^{N_{z,k}}\alpha_{c}^{\zeta +1}\left(z^{\left(i\right)}\right)}{N_{z,k}},c=1,2,...,C \end{array}$$ (14)

$$\begin{array}{c} {\hat{\mu }}_{c}^{\zeta +1}=\frac{\sum_{i=1}^{N_{z,k}}\left(z^{\left(i\right)}\alpha_{c}^{\zeta +1}\left(z^{\left(i\right)}\right)\right)}{\sum_{i=1}^{N_{z,k}}\alpha_{c}^{\zeta +1}\left(z^{\left(i\right)}\right)},c=1,2,...,C \end{array}$$ (15)

$$\begin{array}{c} {\hat{P}}_{c}^{\zeta +1}=\left\{ \begin{array}{c} \frac{1}{k-1}\sum_{i=1}^{k-1}\left({\hat{P}}_{c}^{*}\left(i\right)\right),S\in \text{Encounter}\\ \frac{\sum_{i=1}^{N_{z,k}}\left(\left(z^{\left(i\right)}-{\hat{\mu }}_{c}^{\zeta +1}\right){\left(z^{\left(i\right)}-{\hat{\mu }}_{c}^{\zeta +1}\right)}^{T}\alpha_{c}^{\zeta +1}\left(z^{\left(i\right)}\right)\right)}{\sum_{i=1}^{N_{z,k}}\alpha_{c}^{\zeta +1}\left(z^{\left(i\right)}\right)},\text{otherwise} \end{array}\right. \end{array}$$ (16)

where ζ is the iteration step, $\alpha^{\zeta +1}$ denotes the posterior probability at step ζ+1. And $\alpha^{\zeta +1}$ is estimated by the parameters $\theta^{\zeta }={\left\{{\hat{\pi }}_{c}^{\zeta },{\hat{\mu }}_{c}^{\zeta },{\hat{P}}_{c}^{\zeta }\right\}}_{c=1}^{C}$.

The EM algorithm easily converge to the local optimal solution for it is sensitive to the initial parameters [20,39]. To ensure an optimal result, TSF-GMM-EM uses the predicted location $\mu_{k}^{*}$ as the initialization cluster center after the target trajectory is successfully established. At step ζ+1, the likelihood is updated as


$$\begin{array}{c} L\left({\hat{\theta }}^{\zeta +1}\right)=\sum_{i=1}^{N_{z,k}}L\left({\hat{\theta }}^{\zeta }\right) \end{array}$$ (17)

The iterative process terminates when the log-likelihood difference $\left|ℒ({\hat{\theta }}^{\zeta +1})-ℒ({\hat{\theta }}^{\zeta })\right|$ falls below a predefined tolerance ε. Subsequently, let $\alpha_{c}^{\zeta +1}({𝐳}^{\left(i\right)}|\theta_{EM}^{*})$ represent the posterior probability that measurement ${𝐳}^{\left(i\right)}$ belongs to cluster *c*. The optimal measurement partition is determined by maximizing this posterior probability as follows:


$$\begin{array}{cc} W= & \underset{c}{\text{arg}\max}\alpha_{c}^{\zeta +1}\left({𝐳}^{\left(i\right)}|\theta_{EM}^{*}\right),\\ & i=1,2,...,N_{z,k};\;c=1,2,...,C \end{array}$$ (18)

where *W* denotes the measurements partition results of all measurements, $c_{i}^{*}$ denotes the cluster index of the *i*-th measurement. Accordingly, the final measurement partition set *W* is constructed by grouping all measurements ${𝐳}^{\left(i\right)}$ associated with the same cluster index. These partitioned cells are then incorporated into the GM-PHD filter to update the states and weights of the multiple extended targets.

#### 2.2.2 Intersection situation judgment.

Target tracking algorithms estimate kinematic states and geometric attributes such as shape and orientation of multiple extended targets by establishing consistent associations between measurements and trajectories. Beyond ensuring motion consistency, these trajectories provide critical situational awareness in identifying target proximity and potential intersections. Inspired by the strong short-term spatio-temporal correlations exhibited by extended target motion, the proposed method leverages the feedback from predicted trajectories to quantify the interaction between targets.

Let $G_{k}^{\left(c\right)}$ and $G_{k+1}^{\left(n\right)}$ denote the centroids of the *c*-th measurement set at instant *k* and the *n*-th measurement set at *k* + 1, respectively. To evaluate whether two measurement sets at different time instants originate from the same target, a distance-based metric is employed as follows [40]:


$$\begin{array}{cc} d\left(G_{k}^{\left(c\right)},G_{k+1}^{\left(c\right)}\right)= & \max\left(0,G_{k+1}^{\left(c\right)}-G_{k}^{\left(c\right)}-v_{\max}k\right)\\ & +\max\left(0,-G_{k+1}^{\left(c\right)}+G_{k}^{\left(c\right)}+v_{\min}k\right) \end{array}$$ (19)

where $v_{\max}$ and $v_{\min}$ denote the maximum and minimum velocity of target state change. Generally, the covariance of the zero-mean Gaussian white noise is $Y_{k}$, and the normalized square is denoted as follows:


$$\begin{array}{cc} D\left(G_{k}^{\left(c\right)},G_{k+1}^{\left(c\right)}\right)= & \;d{\left(G_{k}^{\left(c\right)},G_{k+1}^{\left(c\right)}\right)}^{T}\\ & \cdot [{𝐘}_{k}+{𝐘}_{k+1}{]}^{-1}d\left(G_{k}^{\left(c\right)},G_{k+1}^{\left(c\right)}\right) \end{array}$$ (20)

where $D\left(G_{k}^{\left(c\right)},G_{k+1}^{\left(c\right)}\right)$ is random variables with $\chi^{2}$ distribution and the degree of freedom is *p*. In consequence, the deviation threshold φ can be determined in the $\chi^{2}$ distribution table. And $D\left(G_{k}^{\left(c\right)},G_{k+1}^{\left(c\right)}\right)\leq \varphi$ denotes the measurements of the same target at adjacent moments. Therefore, the predicted location $\mu_{k}^{*}$ from the movement estimation could be replaced as follows:


$$\begin{array}{c} \begin{array}{c} \mu_{k}^{*}=\mu_{k-1}^{*}+\frac{1}{k-3}\sum_{k=3}^{k-1}{\left\|{\hat{\mu }}_{k-1}^{*}-{\hat{\mu }}_{k-2}^{*}\right\|}_{2} \end{array} \end{array}$$ (21)

where $\mu_{k}^{*}$ denotes the predicted location of target at instant *k*, and ${\left\|{\hat{\mu }}_{k-1}^{*}-{\hat{\mu }}_{k-2}^{*}\right\|}_{2}$ denotes the euclidean distance between estimated optimal locations of targets at adjacent instants.

The target’s shape at every instant is estimated as:


$$\begin{array}{c} {TR}^{\left(i\right)}=\left[{\hat{\mu }}^{*\left(i\right)};{\hat{P}}^{*\left(i\right)}\right] \end{array}$$ (22)

where ${TR}^{\left(i\right)}$ denoted the i−th target’s trajectory including the location and shape information, and ${\hat{P}}^{*\left(i\right)}$ denotes the i−th target’s shape information at every instant.

Once target’s trajectories are successfully initiated, the possible location of the tracking targets should be the predicted location that estimated from the spatio-temporal domain information


$$\begin{array}{c} {\hat{\mu }}_{c}^{\zeta }={\hat{\mu }}_{k}^{*},S\in \text{Track establishment} \end{array}$$ (23)

Meanwhile, the target’s shape information should remain short-time stationarity when targets meet or approach, therefore, the target being tracked should keep shape characteristics itself before the intersection as follows:


$$\begin{array}{c} {\hat{P}}_{c}^{\zeta +1}=\frac{1}{k-1}\sum_{i=1}^{k-1}{\hat{P}}_{c}^{*}\left(i\right),S\in \text{Encounter} \end{array}$$ (24)

Due to the characteristics of the extended target, the proposed method considers both the location and shape information to judge whether multiple targets encounter occurs. For example, let *t*_1_ and *t*_2_ be two extended target, respectively, and the proposed method judge targets approach or meet as follows:


$$\begin{array}{cc} S= & {\left\|{\hat{\mu }}_{k+1}^{t_{1}}-{\hat{\mu }}_{k+1}^{t_{2}}\right\|}_{2}-\left(\left|\max\sqrt{{\hat{P}}_{k+1}^{t_{1}}}\right|+\left|\max\sqrt{{\hat{P}}_{k+1}^{t_{2}}}\right|\right)\\ & \text{s.t. }\left({\left\|{\hat{\mu }}_{k}^{t_{1}}-{\hat{\mu }}_{k}^{t_{2}}\right\|}_{2}-{\left\|{\hat{\mu }}_{k-1}^{t_{1}}-{\hat{\mu }}_{k-1}^{t_{2}}\right\|}_{2}\right)<0 \end{array}$$ (25)

where $\left|\max\sqrt{{\hat{P}}_{k+1}^{t_{1}}}\right|$ is the maximum value of shape information among target No.1. That the constraint condition is less than 0 means that the two targets are gradually approaching rather than away. Therefore, the proposed method considers that S<0 represents the targets meet.

## 3. Experiment results and discussion

**Fig 1:**
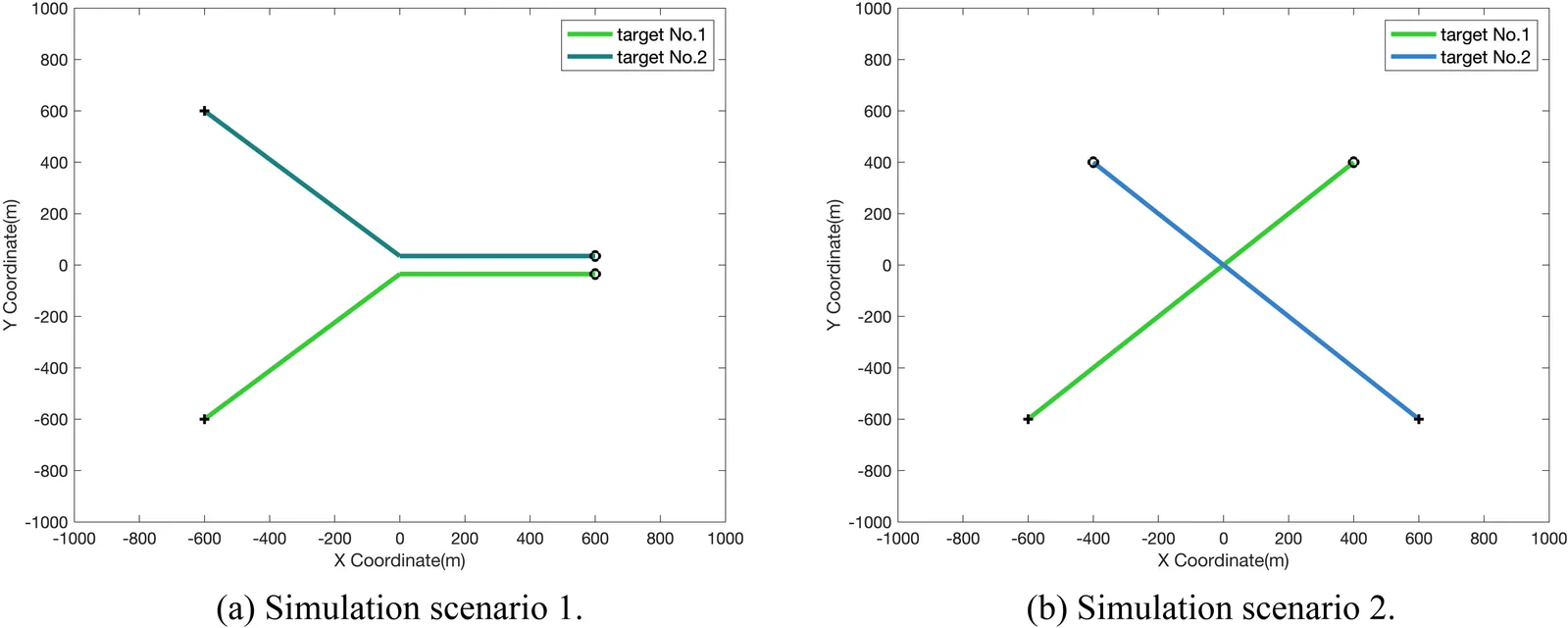
Ground truths of simulation scenarios. (a) Simulation scenario 1. (b) Simulation scenario 2.

This section evaluates the performance of the proposed TSF-GMM-EM method through a comprehensive experimental framework. First, the simulation scenarios and evaluation metrics are described. Subsequently, two specialized experiments are conducted to perform parameter sensitivity analysis and validate the effectiveness of the measurement partitioning algorithm. The overall tracking performance of the proposed method is then verified across two distinct scenarios as shown in Fig 1. All simulations are implemented in MATLAB R2021a and executed on an Apple M1 processor.

### 3.1 Experiment model and parameters

In both simulation scenarios, the number of measurements generated by an extended target is assumed to be Poisson-distributed at each time step, alongside the presence of clutter. The target and measurement states are formulated as follows:


$$\begin{array}{c} x_{k}={\left[x_{k}\;\;y_{k}\;\;v_{k}^{x}\;\;v_{k}^{y}\right]}^{T} \end{array}$$ (26)

$$\begin{array}{c} z_{k}^{\left(i\right)}={\left[x_{k}^{\left(i\right)},y_{k}^{\left(i\right)}\right]}^{T} \end{array}$$ (27)

where $x_{k}$ and $y_{k}$ denotes the truth location of targets at instant *k*, the $x_{k}^{\left(i\right)}$ and $y_{k}^{\left(i\right)}$ denotes the i−th measurements in batches of Cartesian *x* and *y* coordinates.

The state model are established as aforementioned linear Gaussian:


$$\begin{array}{c} x_{k+1}^{\left(i\right)}=T_{k}x_{k}^{\left(i\right)}+G_{k}Q_{k}^{\left(i\right)} \end{array}$$ (28)

where $Q_{k}=\sigma_{v}^{2}I_{2}$ denotes the process noise, and $I_{2}$ is a 2x2 identity matrix, $\sigma_{v}=2\;m/s^{2}$. The parameters of the model are set as:


$$\begin{array}{c} T_{k}=\left[\begin{array}{cccc} 1 & 0 & T & 0\\ 0 & 1 & 0 & T\\ 0 & 0 & 1 & 0\\ 0 & 0 & 0 & 1 \end{array}\right] \end{array}$$ (29)

$$\begin{array}{c} G_{k}={\left[\begin{array}{cc} T^{2}/2 & 0\\ 0 & T^{2}/2 \end{array}\begin{array}{cc} T & 0\\ 0 & T \end{array}\right]}^{T} \end{array}$$ (30)

where *T* is the sampling period, and it is set as one second in these experiments.

The measurements model is also a linear Gaussian model


$$\begin{array}{c} z_{k}=H_{k}x_{k}+e_{k}^{\left(i\right)} \end{array}$$ (31)

where $H_{k}=\left[\begin{array}{cc} 1 & 0\\ 0 & 1 \end{array}\begin{array}{cc} 0 & 0\\ 0 & 0 \end{array}\right]$. And the measurements occur with a ellipsoidal covariance that follows the Gaussian distribution “three-sigma” principle [41], the probability of value distribution in the range [μ−3σ,μ+3σ] is 99.74%.

In the two simulation scenarios, the probability of survival $P_{S}\left(x_{k}^{\left(i\right)}\right)$ and the probability of detection $P_{D}\left(x_{k}^{\left(i\right)}\right)$ for each target is the same as 0.99, and the number of clutter appear in measurement space at every instant follows a Poisson distribution with a mean of 10, i.e., γ=10. The target birth follows a Poisson model with the intensity as follows:


$$\begin{array}{c} v_{b}=\sum_{i=1}^{J_{b}}0.1\cdot N\left(x;m_{b}^{\left(i\right)},K_{b}\right) \end{array}$$ (32)

where $K_{b}=diag\left(\left[100,100,25,25\right]\right)$, $J_{b}$ and $m_{b}$ are different from the three simulation scenarios, then the values are set in the specific scenario, respectively. The pruning threshold is set to $T_{th}=1e-10$, the merging threshold is set to *U* = 8, weight threshold is set to $\omega_{Th}=0.5$ and maximum number of Gaussian terms is set to $J_{\max}=200$ in ET-GM-PHD. The OSPA metric is used to evaluate the tracking performance [42].


$$\begin{array}{cc} {OSPA}_{p,c}(X_{t},{\hat{X}}_{t})= & [\frac{1}{\left|{\hat{X}}_{t}\right|}\left(\min_{\pi \in \Pi_{\left|{\hat{X}}_{t}\right|}}\sum_{i=1}^{\left|X_{t}\right|}d_{c}(x^{i},{\hat{x}}^{\pi (i)}{)}^{p}\right)\\ & {+\frac{c^{p}\cdot (|{\hat{X}}_{t}|-|X_{t}|)}{\left|{\hat{X}}_{t}\right|}]}^{1/p} \end{array}$$ (33)

where *p* and *c* are the order and the cutoff value of OSPA. $d_{c}\left(x,\hat{x}\right)=\min\left(c,d\left(x,\hat{x}\right)\right)$ is the cutoff distance between the cutoff parameter *c* and the euclidean distance between the states x and $\hat{x}$.

### 3.2 Analysis of tracking performance

To evaluate the statistical consistency of the proposed method, 100 Monte Carlo trials are conducted for each simulation. In the following figures, ground truth trajectories are depicted as solid lines, while measurements—comprising both target originated points and clutter are denoted by the “×” marker. Additionally, the “O” notation along the trajectories indicates the spatial extent of the extended targets.

It should be noted that the benchmark methods vary across both scenarios. Specifically, the sub-partition distance method is specifically designed to handle target intersections. In scenarios without intersections, such as Scenario 1, the standard distance partitioning and sub-partition methods exhibit identical tracking performance; therefore, only the primary benchmarks are presented for clarity.

(a) **Simulation scenario 1** $J_{b}$ is set to 2, the parameter $m_{b}$ is set to $m_{b}^{1}={\left[-600,600,0,0\right]}^{T}$ and $m_{b}^{2}={\left[-600,-600,0,0\right]}^{T}$, respectively. The target’s shape are $\sigma_{e_{1}}=\sigma_{e_{1}}=20\;m$, corresponding to the mean of Poisson distribution γ=30. Then, the birth intensity can be calculated as:

$$\begin{array}{c} v_{b}=0.1\cdot N\left(x;m_{b,}^{\left(1\right)},K_{b}\right)+0.1\cdot N\left(x;m_{b,}^{\left(2\right)},K_{b}\right) \end{array}$$ (34)

**Fig 2:**
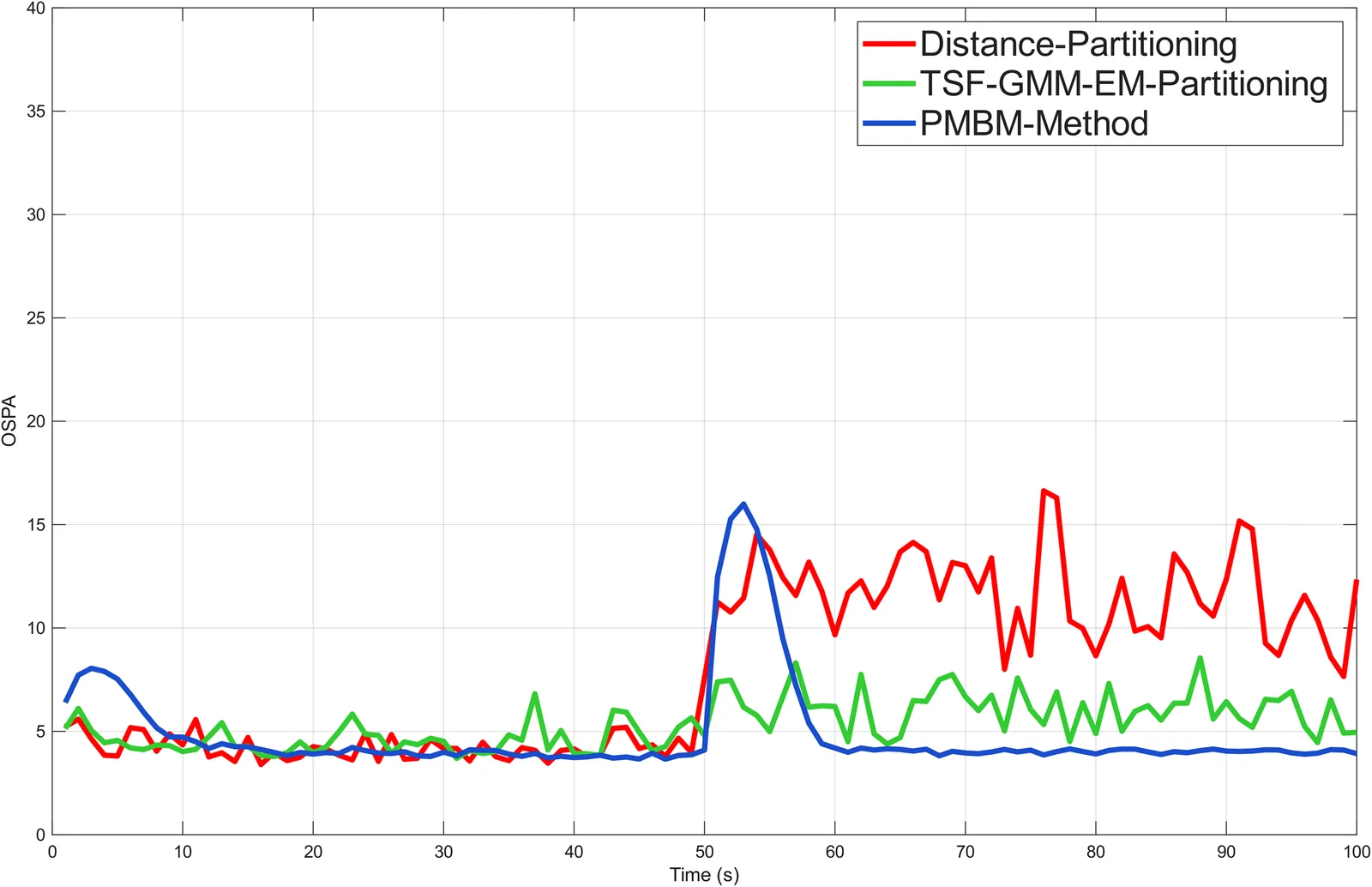
OSPA comparison in simulation scenario 1.

**Fig 3:**
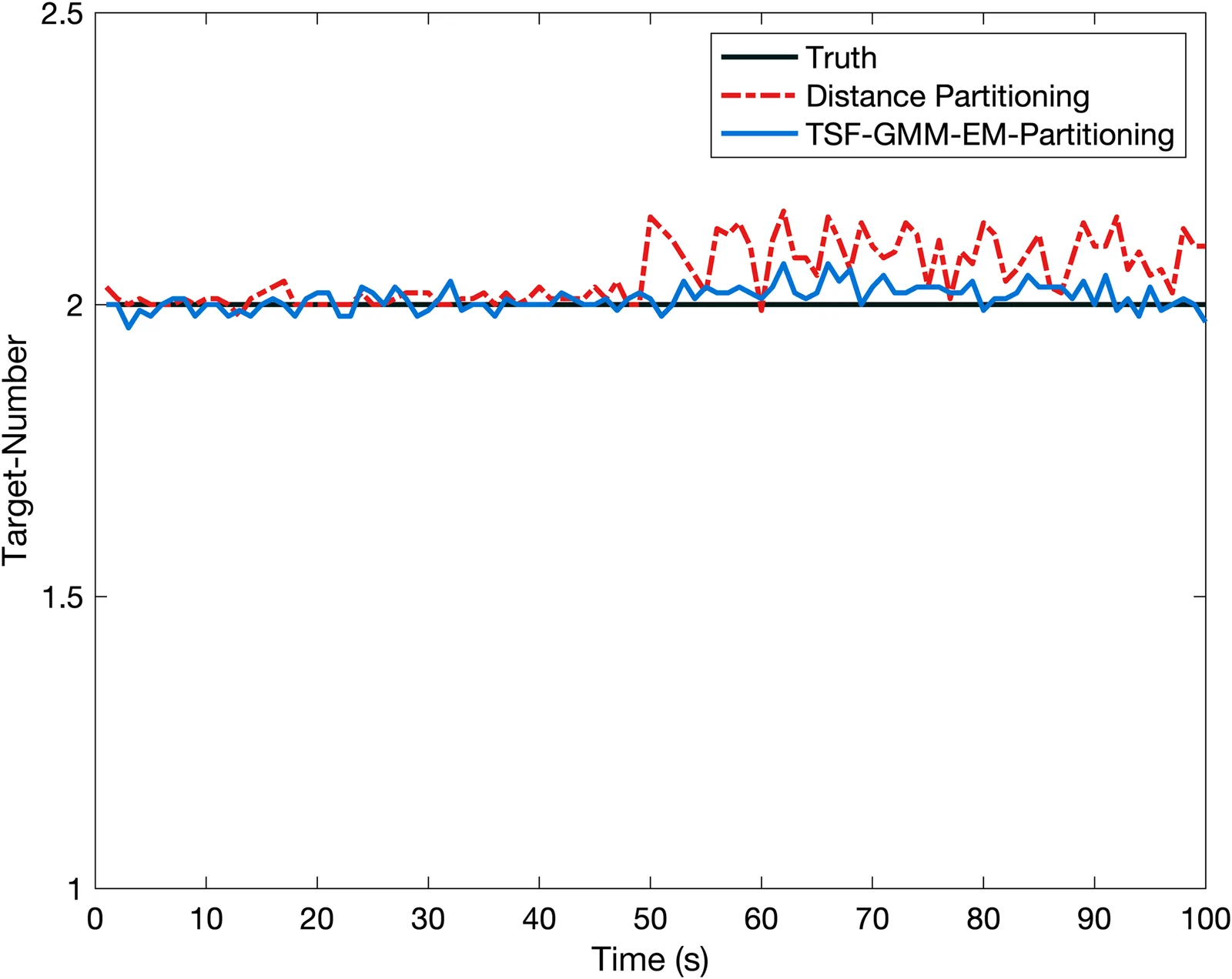
The comparison of number estimation in scenario 1.

Figs 2 and 3 show the OSPA distance and the estimated target number of the evaluated methods over time. As illustrated in Fig 2, during the pre-intersection phase (0–50 seconds), all three methods track the targets accurately, maintaining low and stable OSPA errors.

However, a significant performance divergence occurs during the target crossing interval (50–55 seconds). Due to severe measurement association ambiguity in close proximity, both the Distance-Partitioning method and the PMBM method experience a pronounced error spike, with their OSPA distances climbing up to approximately 15. In contrast, the proposed TSF-GMM-EM-Partitioning framework effectively suppresses this transient error, keeping the peak OSPA distance below 8, which directly demonstrates its superior robustness against intersection ambiguities through the closed-loop trajectory feedback mechanism.

In the post-intersection phase (55–100 seconds), the traditional Distance-Partitioning method fails to recover, exhibiting a persistent and high OSPA error between 10 and 15 due to tracking divergence. Although its steady-state performance is slightly lower than that of the PMBM method, it still significantly outperforms the traditional Distance-Partitioning method, confirming its effectiveness in long-term tracking.

(b) **Simulation scenario 2** The parameters are set to $\sigma_{e_{1}}=15\;m$ and $\sigma_{e_{2}}=6m$, respectively, corresponding to the mean of Poisson distribution $\gamma_{1}=30$ and $\gamma_{2}=15$. $J_{b}$, $m_{b}$ and the birth intensity are the same as in simulation scenario No.1. The two extended targets encounters during the period of 58–64 seconds, TSF-GMM-EM tracks the two targets accurately and continuously under clutter interference when they encounter.

**Fig 4:**
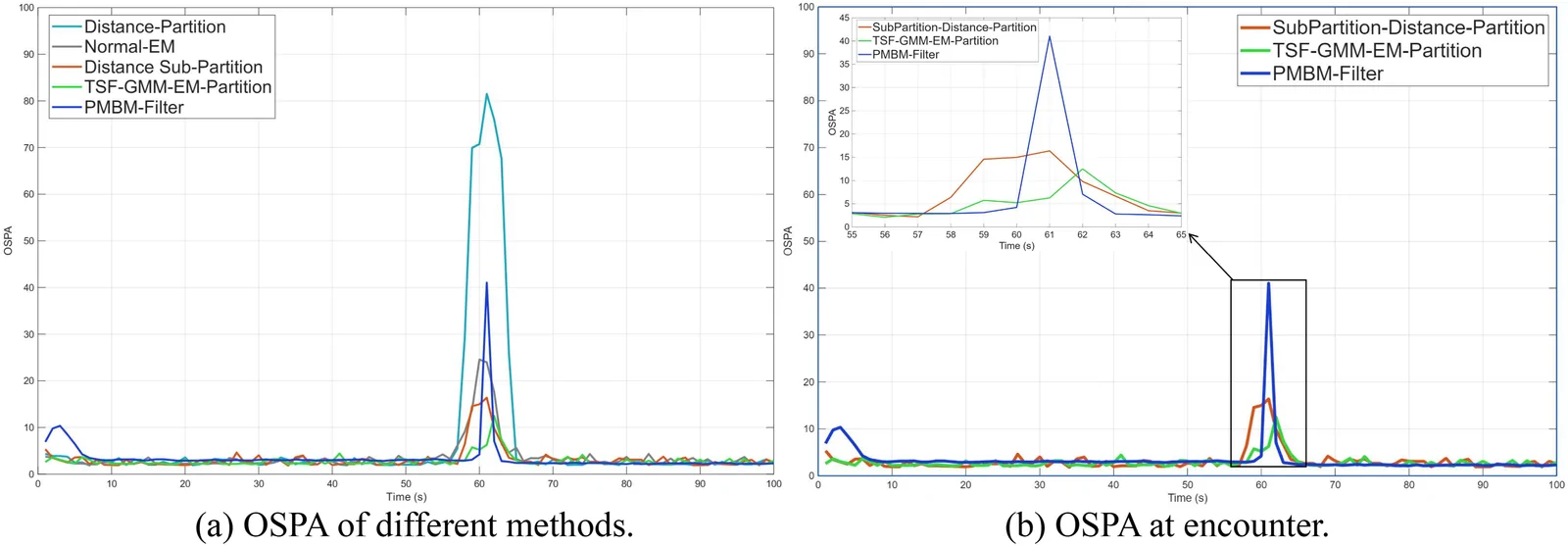
OSPA comparison by different method in simulation scenario 2. (a) OSPA of different methods. (b) OSPA at encounter.

OSPAs estimated by various methods are shown in Fig. 4. The OSPA of normal distance partition method is more than 80 metres, and it loses the targets when they encounters. The reason to failed track target can be attributed to no constraint on the target number. Normal EM method imposes certain constraints on the target number to reduce the tracking errors, however, it just achieve an OSPA of 24.5801 due to the absence of target shape information constraints. Since targets of different sizes produce different numbers of measurements, the existing method calculates the maximum likelihood function to determine sub-partition, then the target might be lost, therefore, this judgment way is not adopted in this paper. However, the target characteristics obtained by the proposed spatiotemporal information feedback are used in the distance sub-partition strategy, the measurement cells exceeding a certain number need to be sub-partition at the intersection moment. The sub-partition distance partition method and TSF-GMM-EM method achieve smaller OSPAs. In order to analyze the tracking performance in detailed, the OSPA curves during the period of 55–65 seconds are magnified and shown in Fig 4(b). During the period of 58–64 seconds, the total OSPAs of sub-partition distance method and TSF-GMM-EM method are 72.2747 and 44.5618, respectively. Constrained by the priori information that the track and the number of multiple targets should be consistent in a short period, TSF-GMM-EM method achieve an OSPA of 5.2318 at 60 second, and it tracks targets more accurately than other methods. The PMBM filter exhibits unstable target tracking at the intersection time of 61 seconds, leading to an increase in OSPA. However, it can quickly adjust and maintain effective tracking in both preceding and subsequent conditions. In the post-intersection phase (65–100 seconds), all methods successfully recover, with their steady-state OSPA errors converging uniformly to a very low level.

**Fig 5:**
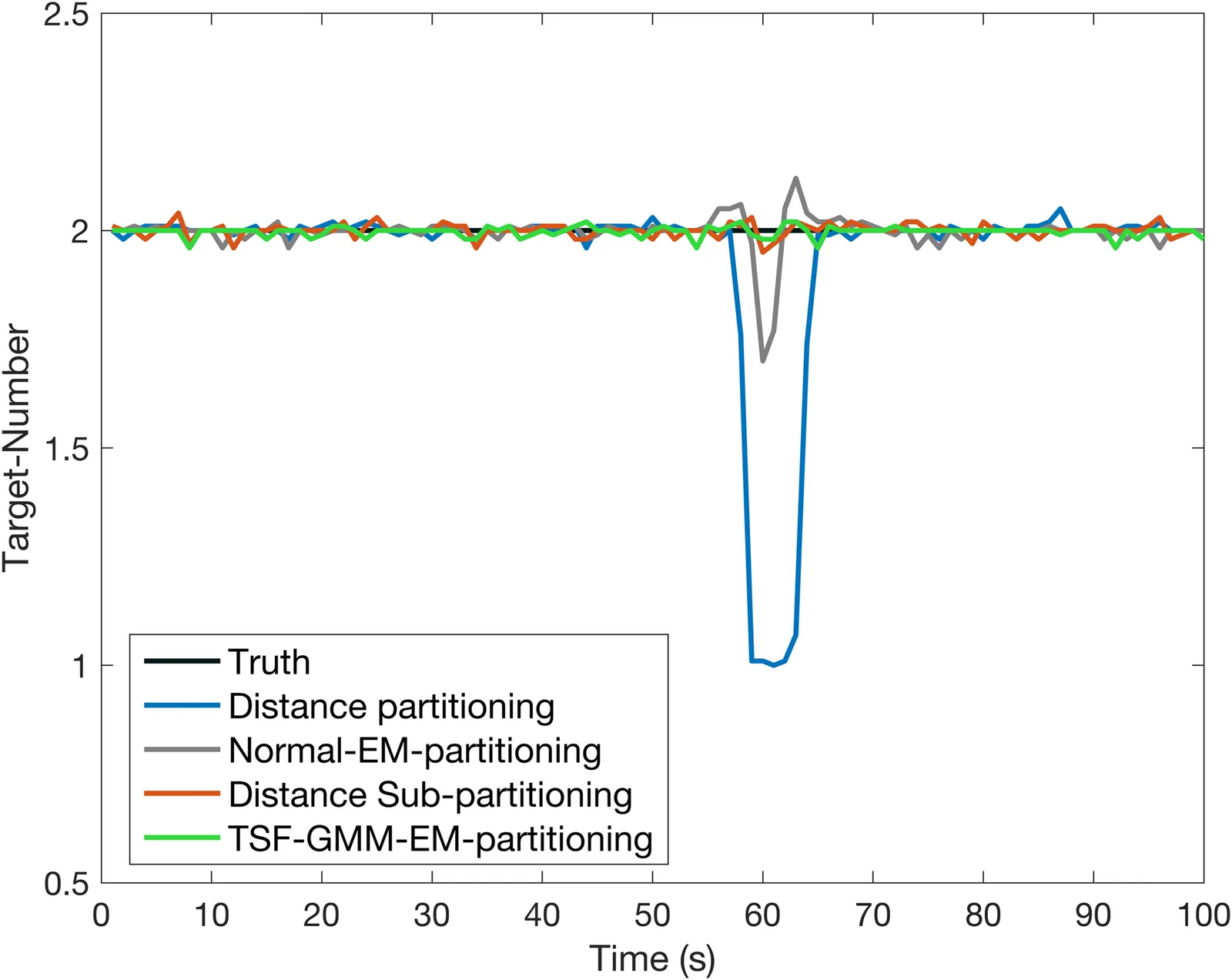
The comparison of number estimation in simulation scenario 2.

The target number estimated by various methods is shown in Fig 5. When the two extended targets encounters, the target numbers estimated by normal distance partition method, normal EM method and TSF-GMM-EM are 1, 1.7 and 1.8, respectively. However, the target number estimated by sub-partition distance partition method and TSF-GMM-EM method is very close to 2, and the estimated fluctuation of the latter is smaller than the former.

### 3.3 Discussion

In this section, two aspects are considered: the robustness and the performance of measurement partition.

1) **Performance of measurement partition**

In order to evaluate the performance of measurement partition algorithm, the measurement partition accuracy is defined as


$$\begin{array}{c} \rho =\frac{S_{N}}{N_{z,k}} \end{array}$$ (35)

where $S_{N}$ represents the cardinality of correctly identified and associated target-originated measurement points, and $N_{z,k}$ denotes the total number of available measurements received by the sensor at time step *k*. The maximum theoretical value of ρ is 1.

**Fig 6:**
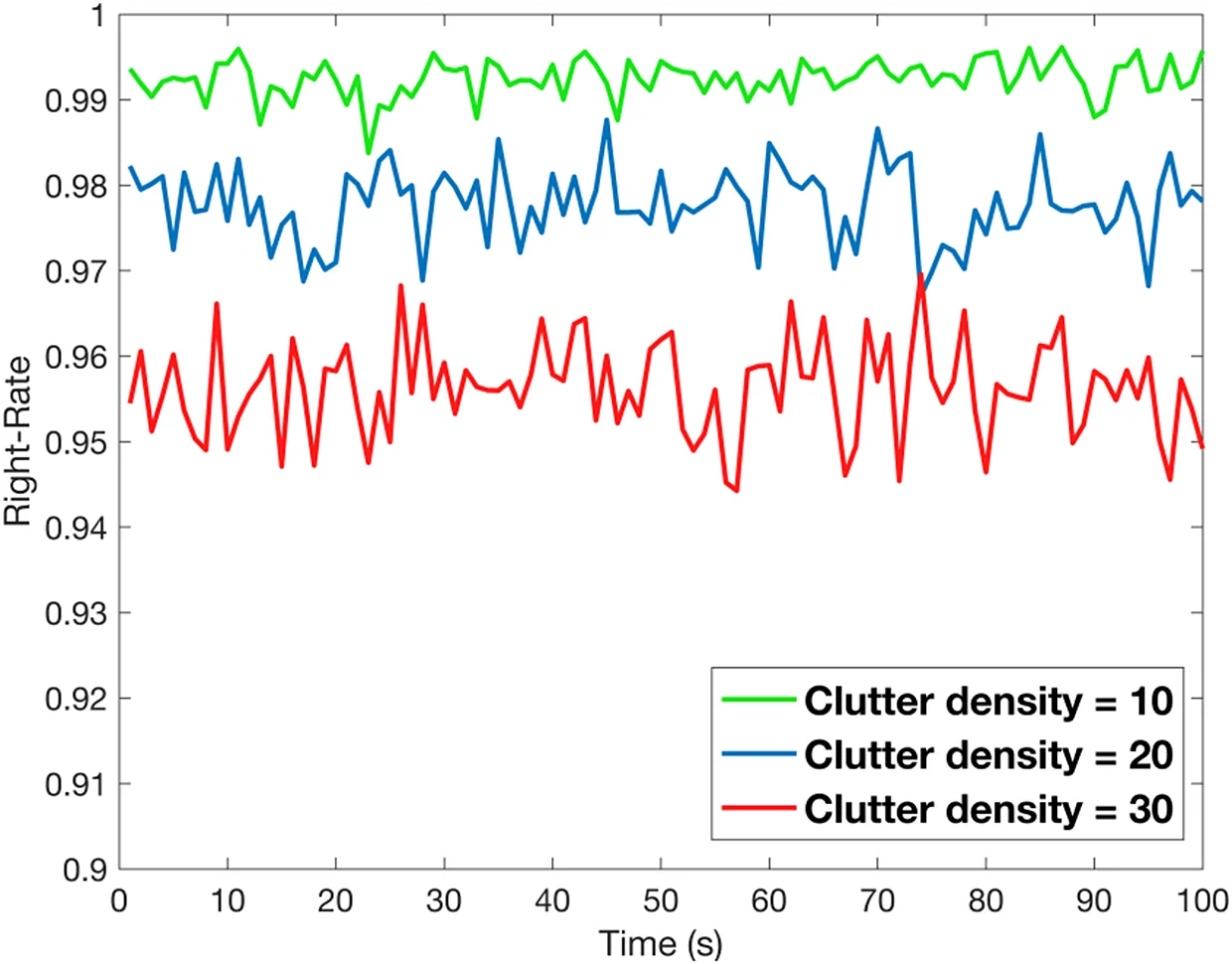
The cluster accuracy of TSF-GMM-EM with different clutter density.

The measurement partition accuracy of 100 Monte Carlo runs is shown in Fig 6. It can be seen that the measurement partition accuracy decrease as the clutter density increases. Nevertheless, the measurement partition accuracy ρ is not lower than 0.946 under the test range of the clutter density, and the proposed method gets outperformance.

In this discussion section, two extended targets are modeled as originating from initial coordinates (244.9 m, 35 m) and (244.9m,−35m), respectively, with their spatial extensions expanding via a Gaussian covariance to an ellipsoidal boundary of $3\sigma_{e_{1}}=3\sigma_{e_{2}}=45\;\text{m}$. To aggressively challenge the algorithm’s resilience against initialization errors, a stochastic perturbation of ±10% is injected into the initial predicted locations. This forces the seeding cluster centers to deviate randomly within the bounded rectangular region of [244.9±10%,35±10%] at the onset of the tracking pipeline.

The empirical partitioning accuracy aggregated over 100 Monte Carlo runs is illustrated in Fig 6. An insightful phenomenon revealed by the curves is that the average clustering accuracy exhibits a slight, monotonic degradation as the ambient clutter density scales up from γ=10 to γ=30. This performance drop is physically attributed to the heightened probability of dense false alarms falling inside the overlapping tracking validation regions, which naturally injects noise into the posterior association weights. Nevertheless, because our trajectory situation feedback loop continuously feeds long-term temporal priors back to anchor the clustering centers, the conventional GMM-EM liability of trapping into uninformative local maxima is completely bypassed. Consequently, the accuracy curves remain remarkably flat and stable across the entire 100-second horizon without suffering from error propagation. Statistically, the partitioning accuracy ρ is strictly maintained above 0.946 even under the harshest clutter condition (γ=30), fully confirming the exceptional spatial-temporal discriminative power of the proposed architecture.

2) **Robustness**

**Fig 7:**
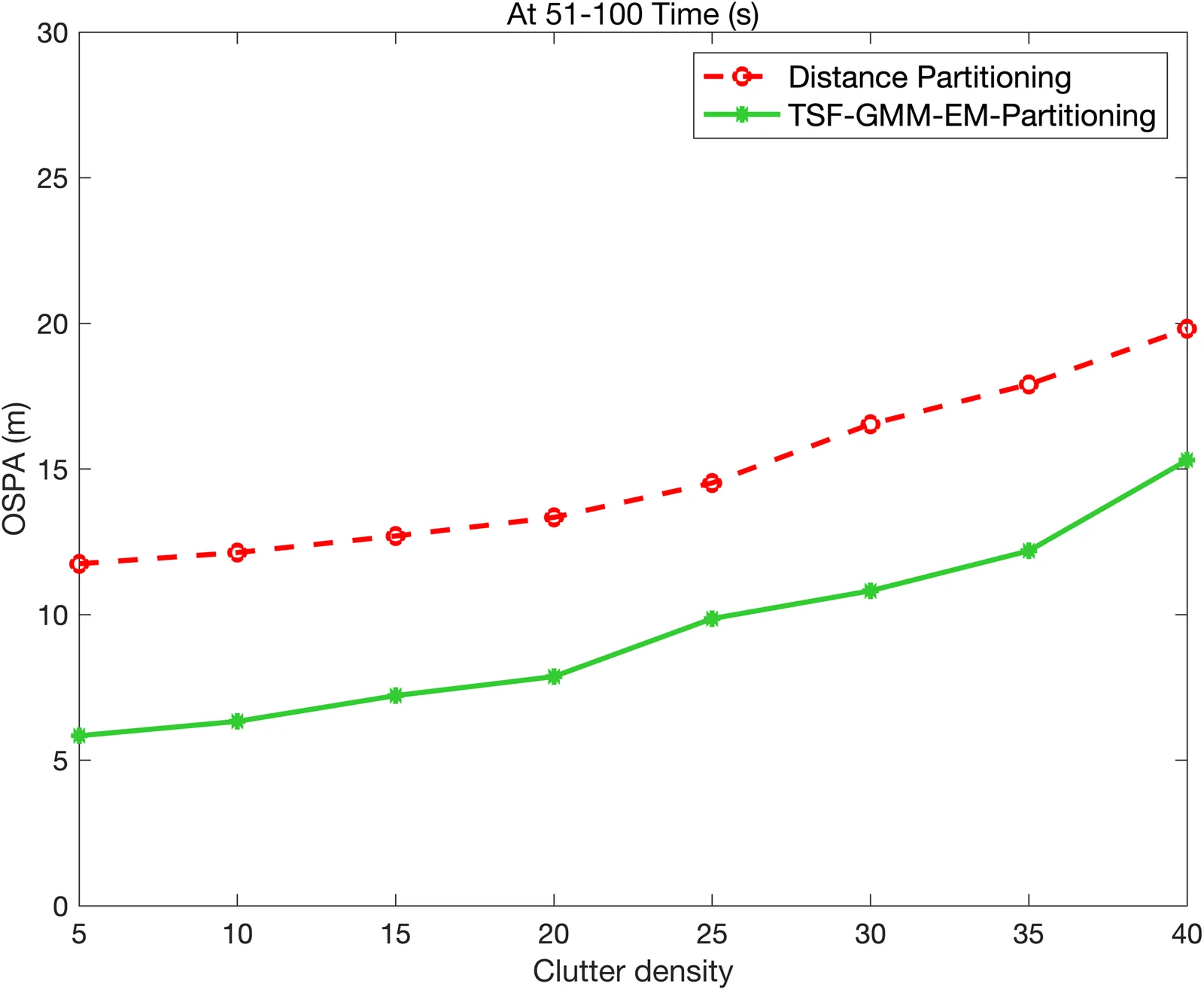
Performance comparison in terms of different clutter rates.

As illustrated by the clutter-sensitivity curves in Fig 7 (compiled over the critical 51–100 seconds interval), a global characteristic shared by both frameworks is that the OSPA distance elevates monotonically as the clutter density increases from γ=5 to γ=40. From a filtering standpoint, this upward trend is mathematically inevitable: an inflation in the false alarm rate forces more clutter points to penetrate the tracking validation gates, which directly corrupts the recursive pseudo-likelihood updates within the GM-PHD filter and biases the centroid estimation.

Nevertheless, the proposed TSF-GMM-EM method demonstrates anti-noise robustness, with its green curve consistently maintaining a lower tracking error than that of the traditional distance partitioning (DP) method across all noise thresholds. When the extended targets closely approach or encounter spatial intersections, the traditional DP method degrades drastically because it operates purely on a single-frame spatial snapshot; lacking cross-frame memory, it easily merges close multi-target point clouds and adjacent clutter into a single cell, leading to track fragmentation. In sharp contrast, by leveraging the predicted states derived from the temporal continuity of target trajectories, our proposed TSF-GMM-EM method enforces strict spatio-temporal consistency constraints onto the mixture model initialization. This closed-loop interaction effectively safeguards the spatial partitioning layer against high-density clutter interference, ensuring that the proposed method achieves robust outperformance in complex noise tracking scenes.

## 4. Conclusion

In this paper, we developed a TSF-GMM-EM method within the framework of the GM-PHD filter to tackle the persistent degradation challenges in multi-extended target tracking. By establishing a closed-loop trajectory feedback framework, this approach successfully realized a bidirectional interaction between cross-frame temporal trajectory evolution and single-frame spatial measurement partitioning, and effectively resolved severe data association ambiguities in dense clutter environments. Furthermore, the introduced prior-guided EM initialization strategy strategically leveraged predicted target kinematic states to seed the soft-clustering process. This mechanism enforced strict spatio-temporal consistency between established trajectories and incoming point clouds, which successfully prevented the conventional GMM-EM pipeline from trapping into uninformative local maxima and drastically mitigated tracking loss or cardinality errors during target intersections. Experimental results demonstrate that the proposed method achieves robust performance in complex tracking scenarios, particularly during target intersections.

Future investigations will focus on expanding the capability of the TSF-GMM-EM approach to accommodate highly non-linear target maneuvers and irregular, complex shape profiles by integrating spatiotemporal Gaussian Processes. Furthermore, we plan to explore alternative probabilistic and optimization paradigms to enrich the theoretical foundations of our framework. Specifically, we aim to extend the current closed-loop filtering structure into a formal Bayesian smoothing framework or a structured MAP estimator with temporal priors to mathematically bound the long-term tracking performance. Investigating variational inference (VI) as an alternative to standard EM partitioning also represents a highly promising avenue, particularly for handling complex scenarios with completely unknown and dynamic target counts through automatic model selection. Additionally, validating the algorithm’s scalability and its adaptive clutter-rate estimation capabilities under heterogeneous clutter profiles using real-world maritime or automotive sensor datasets remains a vital and promising direction for our future research.

## References

[1] Kim J. Tracking multiple underwater targets using adaptive Gaussian mixture probability hypothesis density filter with unknown clutter rate. IEEE Trans Aerosp Electron Syst. 2024;60(6):9154–62. doi: 10.1109/taes.2024.3437343

[2] Indhumathi S, Clement C. Convex-based lightweight feature descriptor for Augmented Reality Tracking. PLoS One. 2024;19(7):e0305199. doi: 10.1371/journal.pone.0305199 39024253 PMC11257345

[3] Liang Z, Liu F, Gao J. Improved GGIW-PHD filter for maneuvering non-ellipsoidal extended targets or group targets tracking based on sub-random matrices. PLoS One. 2018;13(2):e0192473. doi: 10.1371/journal.pone.0192473 29444144 PMC5812665

[4] Zhu Y, Zhou S, Gao G, Zou H, Lei L. Extended emitter target tracking using GM-PHD filter. PLoS One. 2014;9(12):e114317. doi: 10.1371/journal.pone.0114317 25490206 PMC4260874

[5] Guo Y, Li Y, Tharmarasa R, Kirubarajan T, Efe M, Sarikaya B. GP-PDA filter for extended target tracking with measurement origin uncertainty. IEEE Trans Aerosp Electron Syst. 2018;55(4):1725–42.

[6] Li F, Zhou M, Ding Y. An adaptive online co-search method with distributed samples for dynamic target tracking. IEEE Trans Contr Syst Technol. 2018;26(2):439–51. doi: 10.1109/tcst.2017.2669154

[7] Ma Y, Wang Z, Yang H, Yang L. Artificial intelligence applications in the development of autonomous vehicles: a survey. IEEE/CAA J Autom Sin. 2020;7(2):315–29. doi: 10.1109/jas.2020.1003021

[8] Kebria PM, Khosravi A, Salaken SM, Nahavandi S. Deep imitation learning for autonomous vehicles based on convolutional neural networks. IEEE/CAA J Autom Sin. 2020;7(1):82–95. doi: 10.1109/jas.2019.1911825

[9] Kaulbersch H, Honer J, Baum M. EM-based extended target tracking with automotive radar using learned spatial distribution models. 2019 22th International Conference on Information Fusion (FUSION). IEEE; 2019. p. 1–8.

[10] Angelova D, Mihaylova L. Extended object tracking using Monte Carlo methods. IEEE Trans Signal Process. 2008;56(2):825–32. doi: 10.1109/tsp.2007.907851

[11] Koch JW. Bayesian approach to extended object and cluster tracking using random matrices. IEEE Trans Aerosp Electron Syst. 2008;44(3):1042–59. doi: 10.1109/taes.2008.4655362

[12] Zhang H, Ge H, Yang J, Yuan Y. A GM-PHD algorithm for multiple target tracking based on false alarm detection with irregular window. Signal Process. 2016;120:537–52. doi: 10.1016/j.sigpro.2015.10.007

[13] Zhao B, Peng G, Cheng F, Zhang W, Lin H, Wang J. A novel transformer-aided multiple model algorithm for maneuvering target tracking. IEEE Sensors J. 2024;24(23):39578–92. doi: 10.1109/jsen.2024.3482563

[14] Chang W, Chen D, Cao H, Bu L, Wang C, Fu T. Simulation of deep learning-based multitarget track association for ballistic target groups. IEEE Sens J. 2025;25(19):37429–44. doi: 10.1109/jsen.2025.3601590

[15] Qin Z, Liang Y, Li K, Zhou J. Measurement-driven sequential random sample consensus GM-PHD filter for ballistic target tracking. Mech Syst Signal Process. 2021;155:107407. doi: 10.1016/j.ymssp.2020.107407

[16] Wei J, Luo F, Chen S, Qi J. Robust fusion of GM-PHD filters based on geometric average. Signal Process. 2023;206:108912. doi: 10.1016/j.sigpro.2022.108912

[17] Vo B-N, Ma W-K. The Gaussian mixture probability hypothesis density filter. IEEE Trans Signal Process. 2006;54(11):4091–104. doi: 10.1109/tsp.2006.881190

[18] Mahler R. Statistical multisource-multitarget information fusion. Artech; 2007.

[19] Mahler R. PHD filters for nonstandard targets, I: Extended targets. 2009 12th International Conference on Information Fusion. IEEE; 2009. p. 915–21.

[20] Granstrom K, Orguner U. A phd filter for tracking multiple extended targets using random matrices. IEEE Trans Signal Process. 2012;60(11):5657–71. doi: 10.1109/tsp.2012.2212888

[21] Baisa NL, Wallace A. Development of a N-type GM-PHD filter for multiple target, multiple type visual tracking. J Vis Commun Image Represent. 2019;59:257–71. doi: 10.1016/j.jvcir.2019.01.026

[22] Granstrom K, Lundquist C, Orguner O. Extended target tracking using a Gaussian-mixture PHD filter. IEEE Trans Aerosp Electron Syst. 2012;48(4):3268–86. doi: 10.1109/taes.2012.6324703

[23] Akbari B, Zhu H. Tracking dependent extended targets using multi-output spatiotemporal Gaussian processes. IEEE Trans Intell Transp Syst. 2022;23(10):18301–14. doi: 10.1109/tits.2022.3154926

[24] Wahlström N, Özkan E. Extended target tracking using Gaussian processes. IEEE Trans Signal Process. 2015;63(16):4165–78.

[25] Cheng X, Ji H, Zhang Y. Multiple extended target joint tracking and classification based on GPs and LMB filter. IEEE Signal Process Lett. 2024;31:1139–43. doi: 10.1109/lsp.2024.3383954

[26] Yang J, Xiong Y, Cao X, Peng C, Yi W. Joint tracking and classification of vehicles with the PHD filter and Gaussian processes. 2024 27th International Conference on Information Fusion (FUSION). IEEE; 2024. p. 1–7.

[27] Li Z, Zhan R, Zhuang Z. Multiple extended target joint tracking and classification using RMM and TPHD filter. IEEE Signal Process Lett. 2025;32:4164–8. doi: 10.1109/lsp.2025.3625895

[28] Al-Haddad LA, Jaber AA, Dhahir MK, Nagim HY, Algburi ZI. Characterization and prediction of femtosecond laser induced tracks in silver-containing zinc phosphate glass. In: SYSTEM. 2024. p. 10–9.

[29] Li Y, Du Yt, Cui L, Qiu W. Interactive multiple-model probability hypothesis density filter for coexisting point and extended targets tracking. Fifth International Conference on Information Science and Education (ICISE-IE 2025). vol. 13559. SPIE; 2025. 1355917 p.

[30] Qin Z, Kirubarajan T, Liang Y. Application of an efficient graph-based partitioning algorithm for extended target tracking using GM-PHD filter. IEEE Trans Aerosp Electron Syst. 2020;56(6):4451–66. doi: 10.1109/taes.2020.2990803

[31] Sun L, Yu H, Fu Z, He Z, Tao F. Tracking of multiple closely spaced extended targets based on prediction-driven measurement sub-partitioning algorithm. Appl Sci. 2020;10(14):5004. doi: 10.3390/app10145004

[32] Qiu W, Li Y, Cui L, Du Y. A scalable multiple extended targets tracking based on the non-homogeneous poisson process and PHD filter. 2025 17th International Conference on Signal Processing Systems (ICSPS). IEEE; 2025. p. 165–70.

[33] Wang W, Xi J, Zhao D. Learning and inferring a driver’s braking action in car-following scenarios. IEEE Trans Veh Technol. 2018;67(5):3887–99. doi: 10.1109/tvt.2018.2793889

[34] Cao M, Wang R, Chen N, Wang J. A learning-based vehicle trajectory-tracking approach for autonomous vehicles with LiDAR failure under various lighting conditions. IEEE/ASME Trans Mechatron. 2022;27(2):1011–22. doi: 10.1109/tmech.2021.3077388

[35] Aftab W, Hostettler R, De Freitas A, Arvaneh M, Mihaylova L. Spatio-temporal gaussian process models for extended and group object tracking with irregular shapes. IEEE Trans Veh Technol. 2019;68(3):2137–51. doi: 10.1109/tvt.2019.2891006

[36] Granström K, Lundquist C, Orguner U. A Gaussian mixture PHD filter for extended target tracking. 2010 13th International Conference on Information Fusion. IEEE; 2010. p. 1–8.

[37] Wang W, Xi J, Hedrick JK. A learning-based personalized driver model using bounded generalized Gaussian mixture models. IEEE Trans Veh Technol. 2019;68(12):11679–90. doi: 10.1109/tvt.2019.2948911

[38] Li X, Wang W, Roetting M. Estimating driver’s lane-change intent considering driving style and contextual traffic. IEEE Trans Intell Transp Syst. 2019;20(9):3258–71. doi: 10.1109/tits.2018.2873595

[39] Altilio R, Di Lorenzo P, Panella M. Distributed data clustering over networks. Pattern Recognit. 2019;93:603–20. doi: 10.1016/j.patcog.2019.04.021

[40] Zhang C, Li Z, Zhu Y, Luo Z, Qin T. Improved GM-PHD filter with birth intensity and spawned intensity estimation based on trajectory situation feedback control. Remote Sens. 2022;14(7):1683. doi: 10.3390/rs14071683

[41] Jeong J, Park E, Han WS, Kim K, Choung S, Chung IM. Identifying outliers of non-Gaussian groundwater state data based on ensemble estimation for long-term trends. Journal of Hydrology. 2017;548:135–44. doi: 10.1016/j.jhydrol.2017.02.058

[42] Schuhmacher D, Vo B-T, Vo B-N. A consistent metric for performance evaluation of multi-object filters. IEEE Trans Signal Process. 2008;56(8):3447–57. doi: 10.1109/tsp.2008.920469

